# C-955-09. Patient Activity Engagement: Addressing Staff Confidence and Skills

**DOI:** 10.1093/jbcr/irag033.158

**Published:** 2026-04-06

**Authors:** Lisa R Kittleson, Nicole L Rauterkus, Nicholas Larson

**Affiliations:** Regions Hospital Burn Center, Saint Paul, MN; Regions Hospital Burn Center, Saint Paul, MN; Regions Hospital Burn Center, Saint Paul, MN

## Abstract

**Introduction:**

Physical activity for burn patients is beneficial for improving range of motion, strength, overall function, and quality of life. Despite this, evidence suggests that burn patients spend most of their time in bed outside of therapy sessions, even when capable of greater independence. Bedside staff are in a unique position to support patient activity; however, gaps in confidence and skill may limit their active participation. This QI project explored whether implementation of an activity promotion program affected bedside staff confidence and perceived skill in mobilizing patients.

**Methods:**

The burn rehabilitation team at an ABA-verified burn center designed an intervention to improve staff confidence and skill related to patient activity. A pretest survey was administered two months prior to the intervention. Confidence was measured on a 5-point Likert scale (1 = strongly agree to 5 = strongly disagree). A free response question identified specific training needs. The 6-month training intervention included an in-service led by therapists or a recorded version for CME credit. To reinforce content, educational fliers were displayed in staff restrooms, and stoplight-style visual cues were posted in patient rooms to indicate required levels of assistance during activity. A post-test was distributed 6 months later to assess changes. This project was exempt from IRB oversight. Comparisons were done in R using chi-square or Fisher’s exact tests.

**Results:**

Thirty-two clinicians completed the pretest compared to 22 posttest responses. No significant differences in confidence were observed between tests. However, 59.1% of post-test respondents agreed that the activity training directly influenced their clinical practice. Notably, pretest responses emphasized engagement in only basic ADLs, such as commode transfers. In contrast, posttest responses demonstrated greater clinically significant and relevant patient motion, with increased reports of range of motion exercises and chair use. Anecdotally, staff demonstrated more initiative and involvement in activity promotion.

**Conclusions:**

The training resulted in changes in clinical practice despite the lack of significant changes in confidence metrics. The small sample size may have limited power. Barriers identified before the intervention included unfamiliarity with equipment, safe handling techniques, and effective patient encouragement. Structured therapist-led education appeared to address these issues and equipped staff with the tools to engage patients in activity.

**Applicability of Research to Practice:**

Interventions that accommodate diverse learning styles foster staff engagement, collaboration, and functional gains in patients. The results of this project demonstrate that education and skill-building for frontline staff positively impacts clinical practice. Future studies should examine whether this clinical practice change translates to improved patient outcomes.

**Funding for the study:**

N/A.

